# Nanotetrapods: quantum dot hybrid for bulk heterojunction solar cells

**DOI:** 10.1186/1556-276X-8-434

**Published:** 2013-10-19

**Authors:** Furui Tan, Shengchun Qu, Fumin Li, Qiwei Jiang, Chong Chen, Weifeng Zhang, Zhanguo Wang

**Affiliations:** 1 Key Laboratory of Photovoltaic Materials, Department of Physics and Electronics, Henan University, Kaifeng 475004, Henan, People’s Republic of China; 2 Key Laboratory of Semiconductor Materials Science, Institute of Semiconductors, Chinese Academy of Sciences, Beijing 100083, People’s Republic of China

**Keywords:** HBH nanostructure, CdTe NTs, CdSe QDs

## Abstract

Hybrid thin film solar cell based on all-inorganic nanoparticles is a new member in the family of photovoltaic devices. In this work, a novel and performance-efficient inorganic hybrid nanostructure with continuous charge transportation and collection channels is demonstrated by introducing CdTe nanotetropods (NTs) and CdSe quantum dots (QDs). Hybrid morphology is characterized, demonstrating an interpenetration and compacted contact of NTs and QDs. Electrical measurements show enhanced charge transfer at the hybrid bulk heterojunction interface of NTs and QDs after ligand exchange which accordingly improves the performance of solar cells. Photovoltaic and light response tests exhibit a combined optic-electric contribution from both CdTe NTs and CdSe QDs through a formation of interpercolation in morphology as well as a type II energy level distribution. The NT and QD hybrid bulk heterojunction is applicable and promising in other highly efficient photovoltaic materials such as PbS QDs.

## Background

Hybrid bulk heterojunction (HBH) nanostructure is commonly adopted in organic thin film solar cells where excitons are generated first after photon absorption [1,2]. In these photovoltaic devices, the HBH structure enables a highly efficient exciton splitting or charge transferring through an interpenetrated nanoscale heterojunction distributed in the whole active layer. If optimization treatment to phase separation is carried out or efficient photovoltaic materials are adopted, not only the exciton splitting and charge transferring but also charge collection will benefit from the formation of interpenetrated and continuous transportation networks for holes and electrons [3-5]. Being profited from the HBH structure, the efficiency of organic hybrid solar cells has been remarkably improved [2,6,7].

During the research of thin film photovoltaic devices, it was found that HBH structure is not only a patent for organic or organic/inorganic hybrid photovoltaics. Inorganic thin film solar cells based on nanocrystals or quantum dots (QDs) also found their next step to better performance by introducing the HBH nanostructure mentioned above [8]. Recently, it was found that the performance of PbS quantum dot solar cells was remarkably enhanced under a hybrid structure composed of PbS quantum dots and Bi_2_S_3_ nanoparticles [9]. The key factor bringing such an exciting enhancement was attributed to a prolonged charge lifetime which allowed efficient charge separation and transport based on the formation of a nanoscale HBH. Another similar structure was fabricated by infiltrating PbS quantum dots into a porous TiO_2_ layer to form a depleted bulk heterojunction which was found beneficial to exciton splitting [10]. In these devices, an electron donor-acceptor (D-A) model was introduced to discuss the work mechanism of solar cells with a HBH structure. Keeping this in mind, we think that it is reasonable to form interpenetrated and continuous two phases for the highly efficient exciton splitting and charge transportation. For this consideration, a novel HBH nanostructured solar cell was obtained by introducing CdTe nanotetrapod (NT)/CdSe QD hybrids as the photoactive layer and CdTe NTs as the anode buffer layer. Ligand treatment to the bulk heterojunction film composed of NT/QD hybrids ensures an efficient charge transferring and thereafter transporting in interpenetrated pathways. Remarkable photovoltaic performance is obtained with this hybrid composition. The novel HBH structure is commonly applicable and beneficial to other quantum dot-based solar cells with flexible, low-cost, and solution-processable manufacturing process.

## Methods

### Synthesis of CdTe NTs and CdSe QDs

CdTe NTs and CdSe QDs were synthesized according to the procedure in the literature [11] with some modifications. A Cd precursor solution (containing 1 mmol of CdO dissolved in 3 mL of oleic acid (OA) and 3 g tri-*n*-octylphosphine oxide (TOPO)) was heated to 140°C and kept at this temperature for 1 h under nitrogen protection. In another flask, a Te source solution was formed by dissolving 0.5 mmol of Te powder in 3 mL tri-*n*-octylphosphine TOP. The Cd stock solution was heated to 260°C, and then the Te solution was quickly injected. The reaction proceeded for 3 to 4 min at 260°C to produce CdTe nanocrystals with a tetrapod shape. As to CdSe QDs, similar recipe and procedure were used just by replacing Te with 1.0 mmol of Se powder. Both CdTe NTs and CdSe QDs were purified with chlorobenzene/ethanol solvent/antisolvent for at least four times. The final products were dissolved separately in chlorobenzene to form a 40-mg/mL solution.

### Fabrication of solar cells with CdTe/CdSe hybrid bulk heterojunction

The hybrid bulk heterojunction solar cells with a structure of ITO/CdTe/CdTe: CdSe/ZnO/Al was fabricated as follows: firstly, all patterned conductive indium tin oxide (ITO)/glass substrate were ultrasonically cleaned by soap and water, deionized water, acetone, and isopropanol for 15 min, respectively, and then dried at 110°C for 1 h in air. The active layer was produced by spin coating a 30-nm CdTe NTs layer firstly and then seven layers of CdTe/CdSe hybrid. The weight-to-weight ratio of CdTe NTs to CdSe QDs was controlled in the range of 6:1 to 1:2. Following each spin coating, the substrates were heat-treated at 150°C in air (sample A) or solvent treatment using 3-mercaptopropionic acid (MPA)/methanol solution (10% by volume) (sample B). For solvent treatment, two drops of MPA/methanol solution were dispensed onto the CdTe layer or CdTe/CdSe hybrid layer, and the substrate was spun at 2,500 rpm for 15 s after a 6-s wait. Three rinse steps with methanol were applied under the same operation. Afterward, the substrates were annealed at 150°C for 10 min. Finally, a ZnO buffer layer of about 20 nm is formed on the surface of the substrate by spin coating a ZnO quantum dot solution in isopropanol, as was usually done [12]. The solar cell fabrication was finished by thermally depositing a 100-nm aluminum cathode on top.

### Characterization

The shape of CdTe NTs and CdSe QDs was characterized by transmission electron microscopy (TEM) on a Hitachi H-800 (Hitachi High-Tech, Tokyo, Japan) at an acceleration voltage of 80 kV. HBH thin film surface and cross-sectional morphology were measured by field emission scanning electron microscopy (JEOL 7006 F, JEOL Ltd., Tokyo, Japan). Atomic force microscopy (AFM) test was carried out on a Solver P47 SPM (NT-MDT, Moscow, Russia) under semi-contact mode. The crystal structure of hybrid was researched by Raman scattering on a Renishaw RW1000 (Renishaw, Wotton-under-Edge, UK) confocal microscope with a 514-nm line of Ar^+^ iron laser as exciting light. Absorption measurements were carried out on Varian Cary-5000 model (Agilent Technologies, Inc., Santa Clara, CA, USA) UV-visible infrared spectrophotometer. Electrochemical impedance spectra were recorded on a CHI 660E (CH Instruments, Austin, TX, USA) electrochemical workstation. The current–voltage (*I*-*V*) measurements on CdTe/CdSe HBH solar cells are performed on a Keithley 2400 source (Keithley Instruments Inc., Cleveland, OH, USA) in forward bias mode under AM 1.5 (100 mW/cm^2^) illumination. External quantum efficiency (EQE) measurements were carried out on Crowntech test station (Crowntech Inc., Macungie, PA, USA) with a Keithley 2000 multimeter and a standard silicon PV base cell.

## Results and discussion

**Figure 1:**
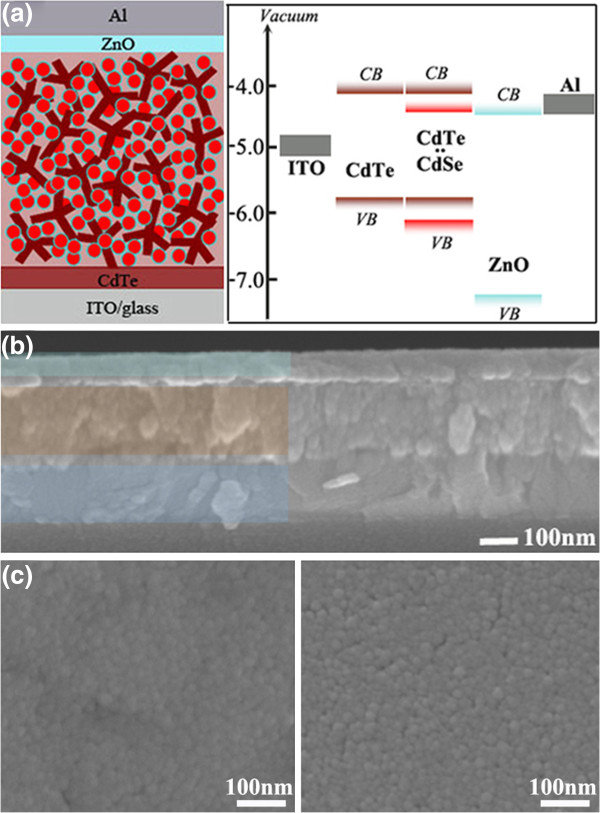
**Hybrid solar cell skeleton, energy level distribution, and SEM images of device and hybrid film surface. (a)** Left: the skeleton of hybrid solar cells in this work, right: the corresponding energy level distribution of the whole device. **(b)** SEM image of the cross section of the device showing the layered structure of the hybrid solar cell (ITO/CdTe/CdTe: CdSe/ZnO/Al). **(c)** SEM image of hybrid film surface without (left) and with (right) MPA treatment.

Figure 1 shows the device structure and the corresponding energy band diagram together with the surface morphology of hybrid films with and without ligand exchange. It mainly contains one donor-acceptor hybrid layer sandwiched between a p-type CdTe NT layer and an n-type ZnO buffer (Figure 1a(left),b). The CdTe NT bottom layer provides a flat contact with the above photoactive layer. In fact, the surface of this buffer layer is not very smooth because of the branch shape of the CdTe nanocrystals. Several reasons are considered for the application of CdTe NTs as a buffer layer in which CdTe would form a cross-linked network. Firstly, just like the CdTe NTs in the hybrid active layer, the same nanocrystal phase and energy level enable the continuous and natural transfer and collection of holes from the active layer to the buffer whose networks are connected at the two layer’s interface. Secondly, the cross-linked network of CdTe NTs in the buffer layer also provides a convenient hole transportation channel to the anode. Furthermore, the CdTe NTs extend their branched arms into the bottom of the active layer so that the contact areas at the interface is enlarged, which correspondingly increases the collection of holes from the active layer. Possibly, this kind of contact interface brings, at the same time, an increased charge recombination due to interface defects. Another optimization of buffer layer materials is however beyond the scope of this work, but it will be our next research focus.

In this work, the CdSe QDs are supposed to fill in the gaps among the branched CdTe NTs. Also, it has suitable conduction and valence band distribution, enabling an effective transfer of holes as well as blocking of electrons. Meanwhile, the type 2 heterojunction at the CdTe/CdSe interface ensures the origin of photovoltaic effect when they are assembled together (Figure 1a(right)). Cross section of the device is shown in Figure 1b from which it is difficult to exactly identify the bottom CdTe NT layer because it has the same crystal phase with that of the above hybrids. The optimized hybrid film thickness is measured to be about 200 nm as seen from the scanning electron microscopy (SEM) image.

**Figure 2:**
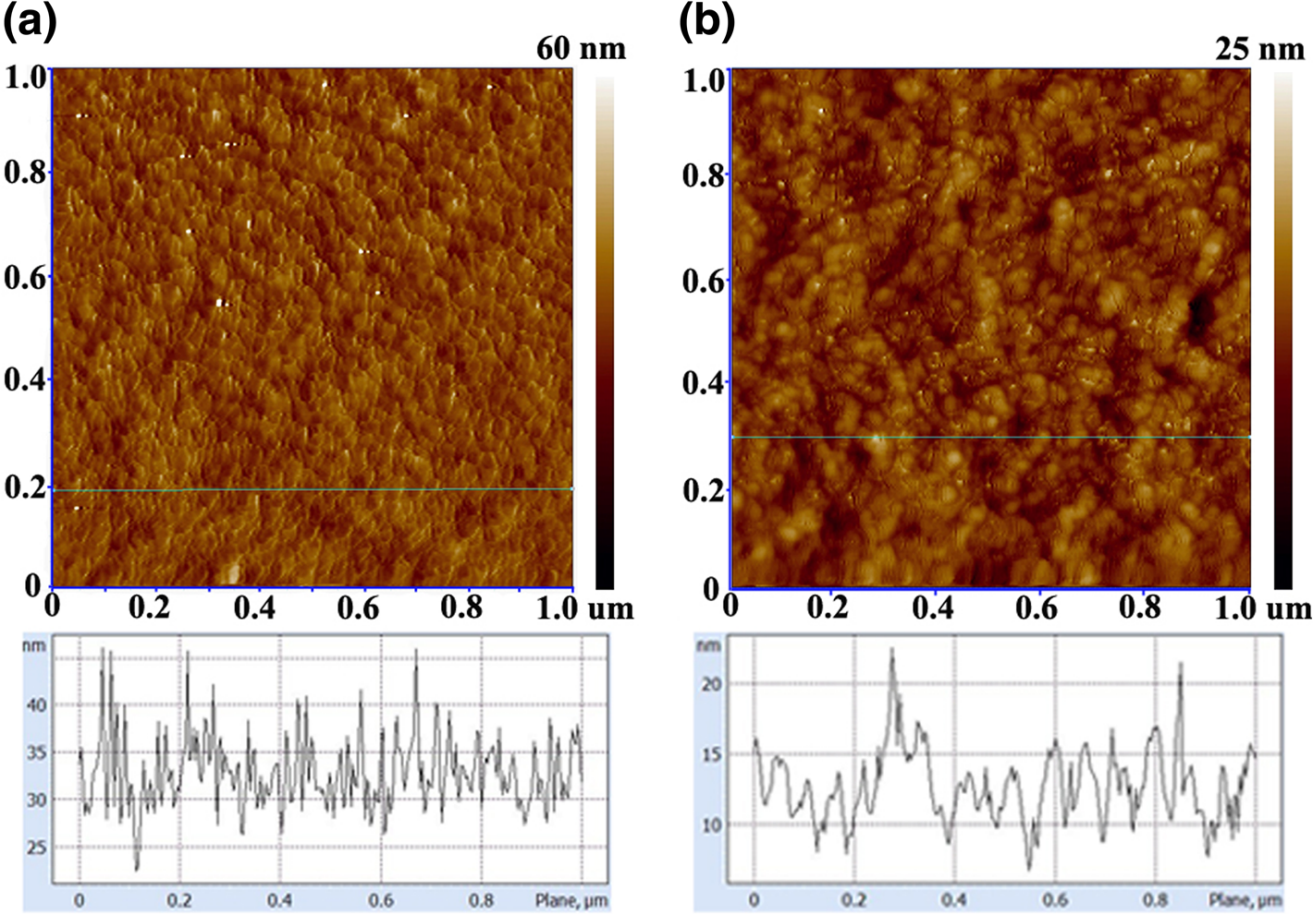
**AFM height images of hybrid films with OA-capped hybrids (a) and after MPA treatment (b).** The bottom images show the corresponding film surface height along the lines in the AFM images.

It was proved that ligand exchange with a short acid molecule is beneficial to a better electric contact between nanocrystals in inorganic QD solar cells [13,14]. In this work, the nanomorphology of the hybrid is critical to the performance of solar cells. A dense contact interface and good interpenetration of the two phases will be expectably beneficial to the performance of inorganic hybrid solar cells. Thus, a comparison of hybrid films with and without MPA treatment was given through SEM characterization in Figure 1c. Densely packed nanocrystal films with homogenous and pinhole-free surface over large areas were observed in both samples. Although there are a few cracks appearing after MPA treatment which is caused by the replacement of a long OA molecule chain, nanocrystal aggregation composed of NTs and QDs is more clearly observed (Figure 1c(right)). The variation in surface morphology after surfactant exchange was also confirmed by AFM characterization in Figure 2.

As can be seen, the OA-capped hybrid nanoparticle thin films exhibit a homogeneous topology, while clusters and agglomerates can be found on the hybrid film after MPA treatment. The surface height along the line part of the AFM image was shown at the corresponding bottom. Mainly, tiny and uniform nanoclusters are observed on the OA-capped hybrid surface, while larger sized nanostructures are demonstrated after MPA treatment, which means that aggregation of nanoparticles appears due to the removal of the long OA surfactant. Thus, ligand exchange correspondingly promotes a closer contact between the two phases from which charge transfer and transportation is benefited.

**Figure 3:**
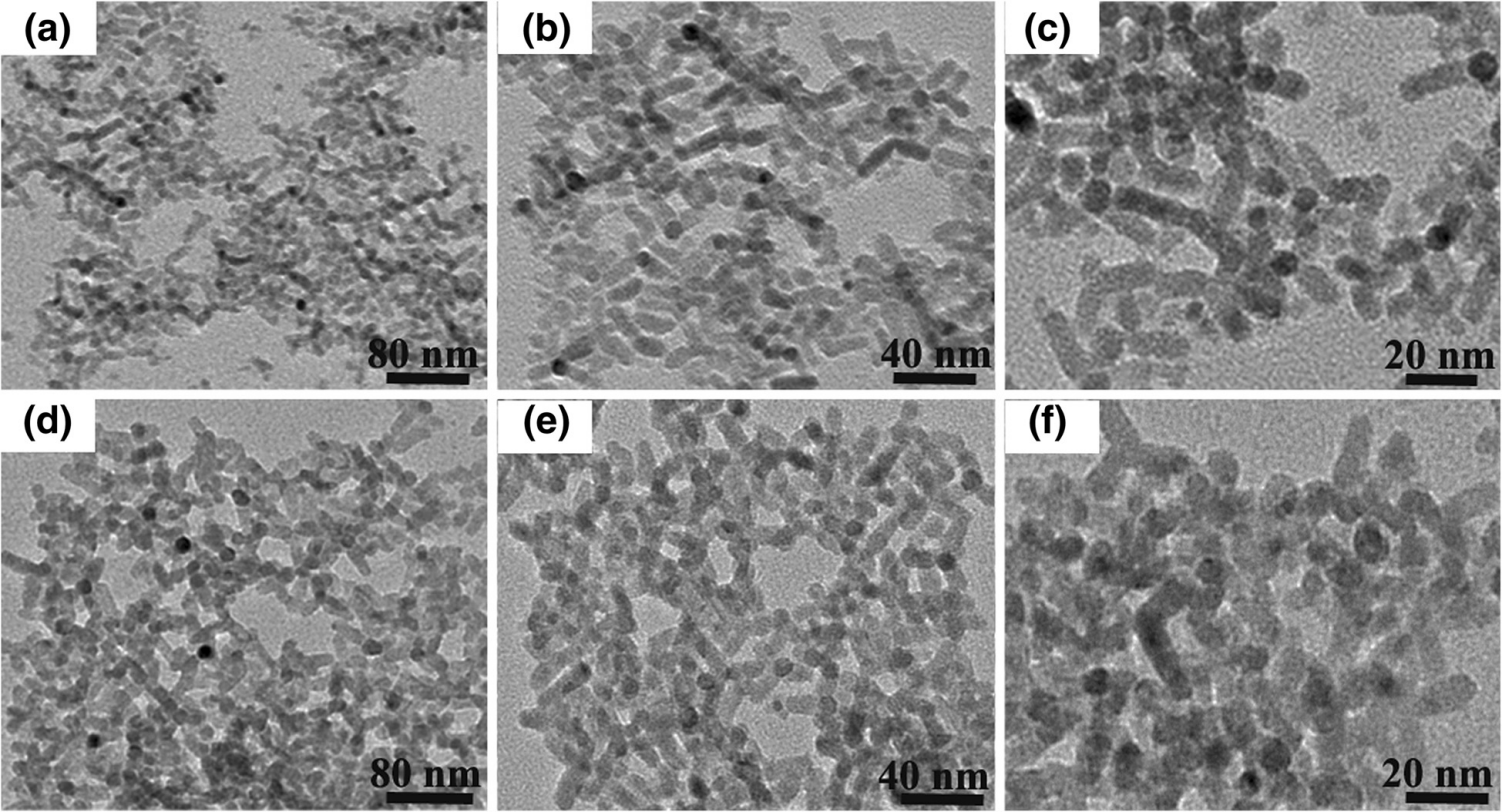
**TEM images of CdTe NT**/**CdSe QD hybrids.** They are prepared by spin coating the hybrid solution on copper net, **(a**, **b**, **c)** without and **(d**, **e**, **f)** with ligand exchange.

In order to more clearly observe the hybrid morphology, TEM thin film samples were prepared by spin coating a diluted hybrid solution onto a fixed copper net. The characterization results are shown in Figure 3. Without MPA treatment (Figure 3a,b,c), the hybrid presents a homogenous connection among NTs and QDs although there are some accumulations due to a large solution concentration (Figure 3a). Self-assembly of nanocrystals can be observed, showing uniform gaps between the adjacent particles (Figure 3b). Especially, the small CdSe quantum dots are presently surrounding and filling the gap of branched CdTe tetrapods (Figure 3c). The obvious self-assembling is caused by the existence of surfactants such as OA or TOPO. In contrast, agglomeration and aggregation in a large scale are shown after the hybrid film was solvent-treated with MPA (Figure 3d). The nanoparticles are densely connected and packed, which makes it difficult to tell where the CdSe quantum dots are located (Figure 3e,f). However, it indeed shows that blending the two kinds of nano-sized building blocks generates a homogenous HBH structure, and MPA treatment to HBH induces denser assembling of the two inorganic phases.

**Figure 4:**
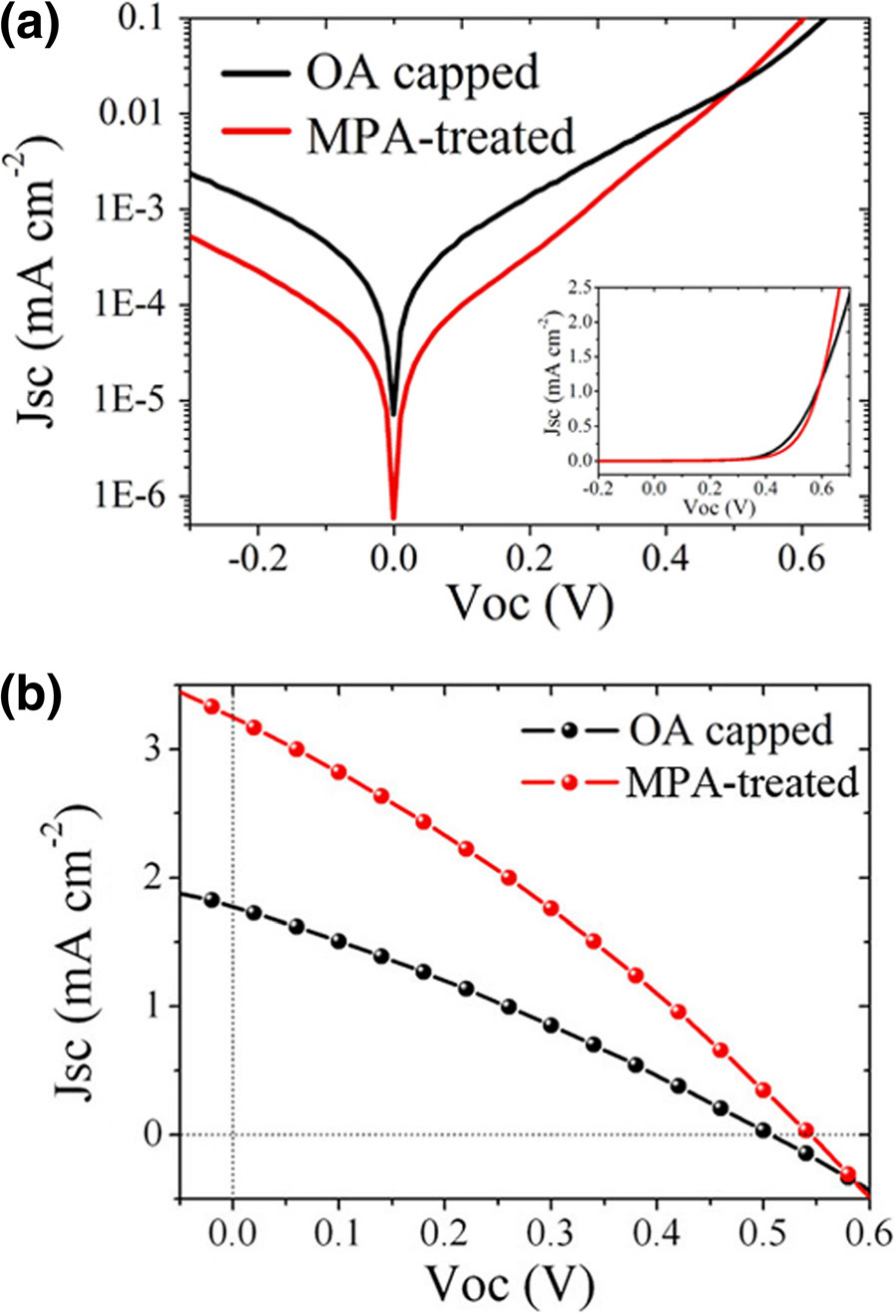
**Current–voltage characteristics of NT**/**QD HBH structured solar cells under (a) dark and (b) 100-mW**/**cm**^**2 **^**illumination.** Data are taken for eight different devices.

Based on the formation of HBH structure, the solar cells were fabricated with the following structure: ITO/CdTe/CdTe: CdSe/ZnO/Al. Firstly, dark *I*-*V* characterization was conducted, and the results were shown in semi-log mode in Figure 4a. A smaller dark current at inverse bias and low forward bias is generated in the MPA-treated solar cells. Besides, an increased diode characteristic is also observable from the dark *I*-*V* curve in the insert of Figure 4a. The corresponding rectifying property is improved due to the enhanced charge collection ability as a consequence of ligand exchange. Figure 4b shows the *I*-*V* characteristics of solar cells under 100-mW/cm^2^ illumination. Improved photovoltaic performance of NT/QD HBH solar cells is obtained after ligand exchange. A drastic increase in *J*_sc_ from 1.8 to 3.3 mA/cm^2^ enables efficiency enhancement from 0.26% to 0.53%. Besides, a slight increase in FF and *V*_oc_ is also found after MPA treatment of the NT/QD solar cells.

**Figure 5:**
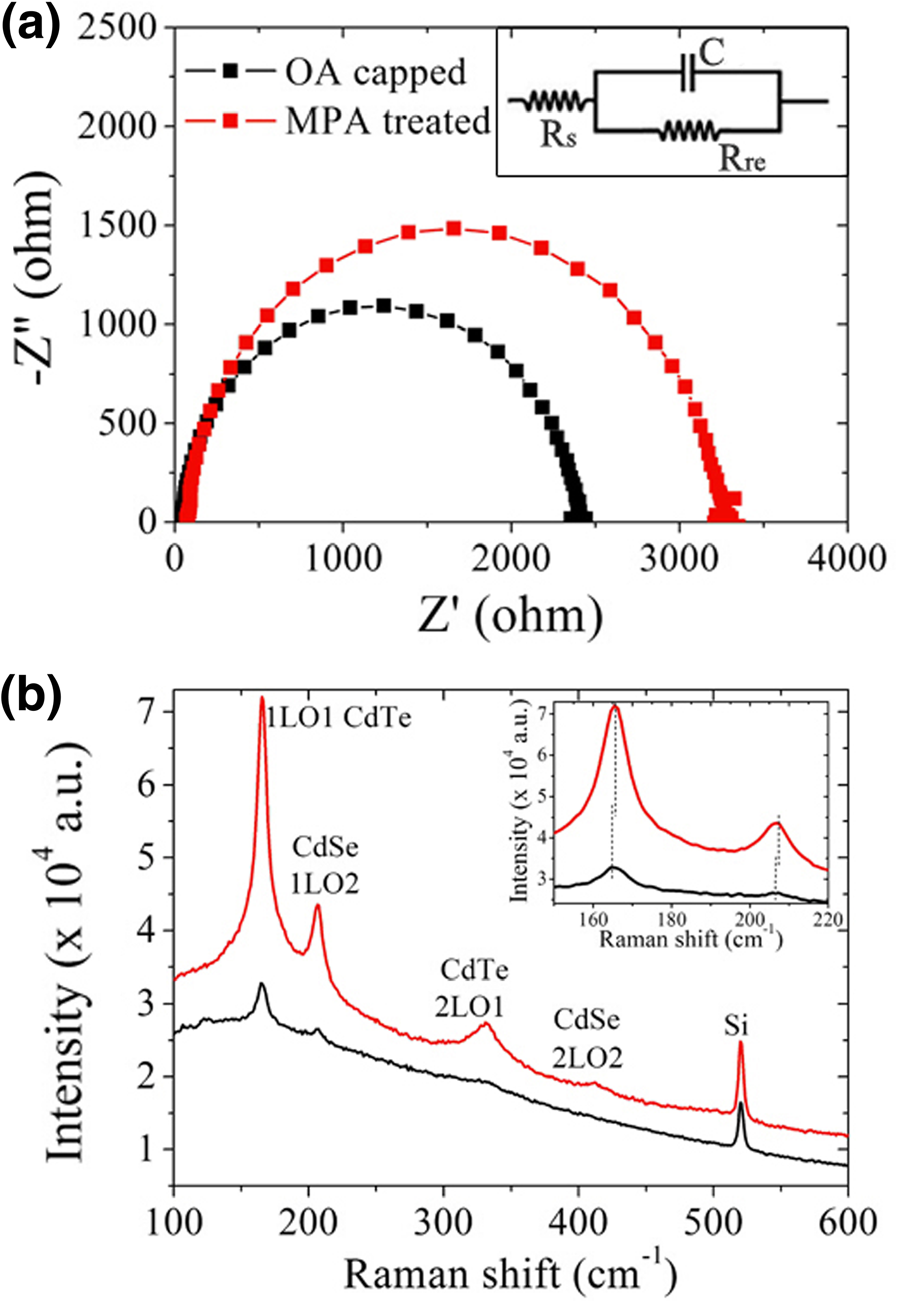
**Electrochemical impedance and Raman spectra of HBH solar cells and film.** Electrochemical impedance spectrum of CdTe NT**/**CdSe QD HBH solar cells **(a)** and Raman spectrum of NT**/**QD HBH film **(b)**. The insert in **(b)** shows the enlarged signals from 150 to 220 cm^-1^.

In order to access the influence of ligand exchange on the performance of NT/QD HBH solar cells, electrochemical impedance spectroscopy (EIS) was used to analyze the dynamic behavior of charge transportation (Figure 5). One semicircle with a frequency variation mainly from 100 to 10 KHz is observed in the Nyquist plot of each solar cell. This frequency response is correlated with a charge transfer process that occurred at the CdTe/CdSe hybrid interface [15,16]. Thus, an equivalent circuit with just one parallel component is given in the insert of Figure 5a, in which *R*_s_ represents the series resistance, *R*_re_ is the charge transfer recombination resistance, and *C* is the capacitance. The Nyquist plot has an enlarged semicircle diameter after ligand exchange, which indicates an increased electron recombination resistance (*R*_ct_) [17,18]. Besides, the effective recombination rate constant (*k*_eff_), which is estimated to be equal to the peak frequency (*ω*_max_) of this arc [15,19], is a little smaller in the MPA-treated NT/QD HBH solar cell than that in the OA-capped hybrids. Thus, the electron lifetime (*τ*) evaluated as *τ* = 1/2*πω*_max_ is accordingly increased after MPA treatment. A larger *R*_ct_ as well as *τ* value means a smaller leakage current and reduced charge trapping, elucidating the smaller dark current at inverse bias and low forward bias in Figure 4a. The decreased charge recombination as well as increased lifetime will also improve the charge transfer efficiency between adjacent NTs and QDs through reducing electron localization or trapping in long insulated OA-capped nanoparticles so that an enlarged *J*_sc_ is obtained. The reason for a slight increase in FF and *V*_oc_ is also mirrored from the EIS result here.

Raman spectrum is a useful tool as it provides short-ranged microstructure information that is further helpful to understand the electric behavior in the EIS result. As shown in Figure 5b, compared with the OA-capped HBH film, both the first and the second longitudinal optical phonon mode of CdTe can be observed around 165 cm^-1^ (1LO1) and 330 cm^-1^ (2LO1) after the NT/QD HBH film was treated with MPA (sample B). The same phenomenon happens with CdSe. The enhancement in Raman peak intensity was suggested to be correlated with molecule adsorption (with large polarity such as this) that induced the passivation of surface states [20-22]; herein, there was an adsorption of MPA on the surface of CdTe NTs and CdSe QDs through Cd-S bond which reduces the electron trapping state caused by the Cd dangling bond. This correspondingly results in a decreased charge trapping and recombination rate, as exhibited from the EIS analysis in Figure 5a. Interestingly, a slight blueshift of the 1LO1 mode from CdTe and 1LO2 mode from CdSe can be observed after MPA treatment, which, in accordance with TEM characterization in Figure 3, indicates a more densely packed microstructure in the hybrid film [23].

**Figure 6:**
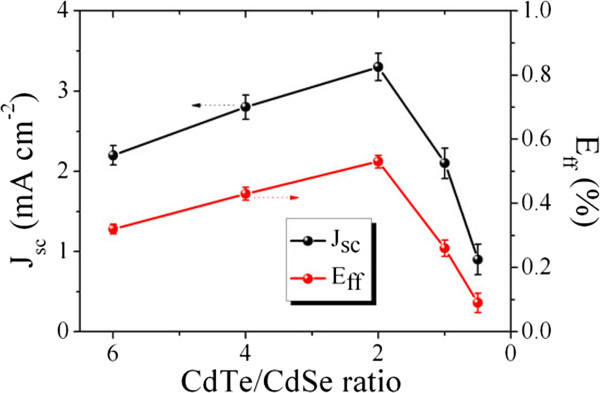
**The effect of CdTe NT**/**CdSe QD mass ratio on HBH solar cell characteristics.**

Figure 6 shows the *J*_sc_ and *E*_ff_ dependence on the mass ratio of CdTe NTs to CdSe QDs. The maximum *J*_sc_ is found to be at an optimum ratio of 2:1, beyond which the *J*_sc_ value drastically decreases due to a relative lack of photoactive CdTe. The variation of *E*_ff_ is mainly dominated by *J*_sc_, reaching a remarkable value of 0.53% at 2:1. Note that this optimum mass ratio is much larger than that in the research with both spherical-shaped nanoparticles [9]. It is easily understandable that the mass of one CdTe nanotetrapod is several times larger than that of one CdSe quantum dot; the optimized CdTe/CdSe ratio ensures a suitable quantity of CdSe QDs surrounding one CdTe nanotetrapod so that a continuous percolation of both CdTe and CdSe is achieved. In this way, efficient charge extraction is allowed by virtue of the interpenetrated donor-acceptor networks.

**Figure 7:**
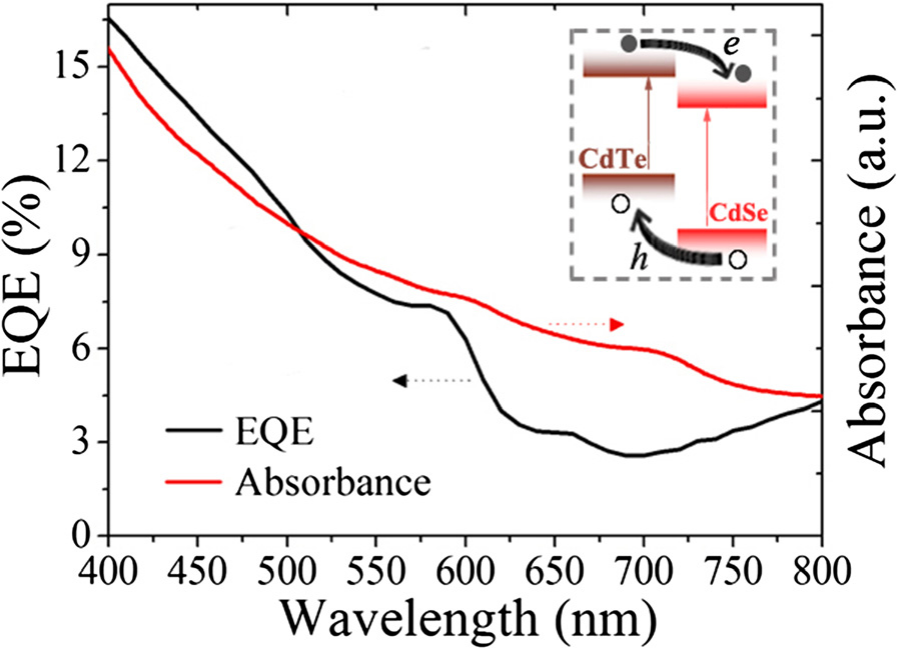
**External EQE and absorption spectrum of NT/QD HBH solar cells.** The insert shows the energy level diagram at the CdTe/CdSe interface and the corresponding charge transfer process.

In order to evaluate the NT/QD hybrids in facilitating the device’s energy conversion efficiency, a direct comparison of EQE and light absorption of solar cells was carried out, and the result is shown in Figure 7. The hybrid thin film device shows a combined light absorption of CdSe and CdTe nanoparticles with peaks near 600 and 700 nm, respectively. An observable similarity in curve shape is found in the EQE result. This phenomenon suggests that by forming a HBH nanostructure, both CdTe NTs and CdSe QDs make their contribution to the total photocurrent. A D-A model is applicable to the operation mechanism of NT/QD hybrids (Figure 7 insert). In the hybrids, CdTe NTs play a role of the electron donor as well as hole acceptor while CdSe QDs as electron acceptor and hole donor. Based on this model, the shapes of branched CdTe and spherical CdSe nanoparticles expectably facilitate the interpenetration of D-A networks which is desired in highly efficient HBH solar cells. This novel HBH structure is commonly applicable in other photovoltaic devices based on nanocrystals such as the efficient PbS QD solar cells. Further research on performance improvement of PbS QD solar cells with a NT/QD HBH structure is under way.

## Conclusions

In conclusion, an efficient solar cell based on an all-inorganic HBH nanostructure composed of NTs and QDs is introduced. Both the CdTe NTs and CdSe QDs make a contribution to photovoltaic performance through their respective photoelectric response region. The interpercolated and continuous networks of CdTe NTs (as electron donor and hole acceptor) and CdSe QDs (as electron acceptor and hole donor) are a critical access in achieving a highly efficient charge transfer and transport. Ligand exchange process enables compacted contact between NTs and QDs which boosts the infiltration of CdSe QDs into the branched CdTe NTs and therefore enhances charge transfer at the heterojunction interfaces. This novel hybrid nanostructure will allow further improvement in photovoltaic performance of the efficient PbS QD solar cells, which is more interesting and exciting.

## Competing interests

The author(s) declare that they have no competing interests.

## Authors’ contributions

FRT carried out the synthesis and fabrication experiments and drafted the manuscript. SCQ and WFZ participated in the sequence alignment. FML carried out the SEM and Raman characterization experiments. CC and QWJ conceived the study and participated in its design. ZGW participated in the design of the study and performed the analysis. All authors read and approved the final manuscript. All authors read and approved the final manuscript.
